# The Impact of Participation in the Parkinson's Pals Program on Psychosocial Symptoms in Parkinson's Disease: An Unblinded Feasibility Study

**DOI:** 10.1002/mdc3.70589

**Published:** 2026-04-01

**Authors:** Jaskeerat Gujral, Udit Garg, Om H. Gandhi, Lynn Eickholt, Whitley W. Aamodt

**Affiliations:** ^1^ Department of Bioengineering University of Pennsylvania School of Engineering & Applied Science Philadelphia Pennsylvania USA; ^2^ Department of Neuroscience University of Pennsylvania School of Arts & Sciences Philadelphia Pennsylvania USA; ^3^ Department of Neurology University of Pennsylvania Perelman School of Medicine Philadelphia Pennsylvania USA; ^4^ Translational Center of Excellence for Neuroepidemiology and Neurology Outcomes Research University of Pennsylvania Perelman School of Medicine Philadelphia Pennsylvania USA; ^5^ Leonard Davis Institute of Health Economics University of Pennsylvania Philadelphia Pennsylvania USA

**Keywords:** Parkinson's disease, loneliness, demoralization, quality of life

## Abstract

**Background:**

Persons with Parkinson's disease (pwPD) frequently experience psychosocial symptoms that are inadequately addressed by traditional clinical approaches.

**Objectives:**

To evaluate the impact of Parkinson's Pals, a student‐led virtual, intergenerational program, on psychosocial symptoms in pwPD and student knowledge.

**Methods:**

PwPD were paired with undergraduates for eight virtual meetings over  four months. Scores on the UCLA Loneliness Scale (UCLA‐LS), Kissane Demoralization Scale (DS), Scales for Outcomes in Parkinson's Disease‐Psychosocial Functioning (SCOPA‐PS), Parkinson Disease Questionnaire‐39 (PDQ‐39), and student knowledge self‐assessments were compared before and after program participation.

**Results:**

Twenty‐five pwPD‐student pairs participated. There was significant improvement in median total UCLA‐LS (*p* = 0.031) and DS (*p* < 0.001) scores but not SCOPA‐PS or PDQ‐39 scores (*p* > 0.05). Students also reported improved familiarity with PD (*p* < 0.001).

**Conclusions:**

Participation in Parkinson's Pals was feasible, reduced loneliness and demoralization in pwPD, and enhanced student education. Further studies are needed to explore the psychosocial benefits of intergenerational programs for pwPD.

Parkinson's disease (PD) is characterized by heterogeneous motor and non‐motor symptoms that significantly diminish quality of life (QoL).^1^ Persons with PD (pwPD) also experience hypomimia, hypophonia, and gait dysfunction that directly limit social engagement.^2^, ^3^, ^4^, ^5^, ^6^ Consequently, many pwPD experience loneliness and social isolation.^7^, ^8^, ^9^ A prior study found that loneliness occurs in 24–54% of pwPD, which exceeds rates of loneliness in healthy, age‐matched peers.^10^ Social disconnection also contributes to demoralization, a syndrome characterized by feelings of helplessness, hopelessness, and meaninglessness that affects between 25% and 30% of pwPD and overlaps with depression.^11^, ^12^, ^13^

Although traditional clinical approaches insufficiently address psychosocial symptoms in pwPD, prior research suggests that new relationships may improve psychosocial well‐being and physical health in older adults.^14^ Several groups have developed intergenerational programs to promote social connectedness among persons with neurodegenerative diseases, including the Time for Dementia Programme^15^ and University of Louisville Parkinson's Disease Buddy Program.^16^ Despite their benefits, both programs require in‐person meetings and may overlook homebound individuals who are in greatest need of social connection.

In 2021, undergraduates at the University of Pennsylvania (Penn) developed Parkinson's Pals (PD Pals), an intergenerational program that pairs undergraduate students with pwPD for structured 1‐on‐1 conversations through virtual platforms.^17^ The impact of participation on psychosocial functioning in pwPD remains unknown, and we conducted a unblinded feasibility study to determine the influence of participation on validated measures of loneliness, demoralization, social functioning, and disease‐specific QoL. Our secondary objective was to assess the program's impact on student knowledge. We hypothesized that regular social contact with young adults would reduce loneliness and improve demoralization, social functioning, and QoL in pwPD, while simultaneously educating students.

## Methods

### Protocol Approval

This study was approved by the Penn Institutional Review Board. All participants provided written informed consent.

### Recruitment

PD Pals, a student‐led non‐profit organization, pairs undergraduate students with pwPD to promote social connectedness.^17^ Pairs meet virtually for 1 h every 1–2 weeks each semester to discuss mutually chosen topics, including hobbies and career aspirations. Students are recruited via word‐of‐mouth and university flyers. Eligible students are 18 or older, have no disciplinary history, commit 1 h/week for 4 months, and successfully complete a phone interview with program leaders assessing their communication skills and motivation. This screening process ensures that student participants possess the qualities necessary for meaningful relationship development. Program leaders also complete PD training through the Davis Phinney Foundation and serve as a resource for others. PwPD are referred by their neurologist or approached by program leaders during routine visits at the Penn Parkinson's Disease and Movement Disorders Center. Eligible pwPD are 18 or older and proficient in English, have clinically diagnosed PD, and have decision‐making capacity as judged by their treating physician. No persons with dementia are referred for participation.

From September 2023 to July 2025, all students and pwPD who enrolled in PD Pals were offered optional participation in the unblinded feasibility study. Interested participants were consented and completed pre‐program assessments described below. After 8 virtual sessions over a 4‐month semester (approximately 2 sessions/month), participants completed post‐program assessments.

### Study Outcomes

We administered 4 patient‐reported assessments: UCLA Loneliness Scale (UCLA‐LS),^18^ Kissane Demoralization Scale (DS),^19^ Scales for Outcomes in Parkinson's Disease‐Psychosocial Functioning (SCOPA‐PS),^20^ and Parkinson Disease Questionnaire‐39 (PDQ‐39).^21^ These assessments are validated for use in pwPD,^11^, ^13^, ^22^ and complete descriptions, scoring instructions, and minimal clinically important differences (MCID) are summarized in the Supplemental Methods. There is no established MCID for the UCLA‐LS or DS. To determine whether participation in PD Pals improved student knowledge of PD, we also administered a pre‐ and post‐program knowledge survey. All participants completed a demographics form and post‐program satisfaction survey.

### Statistical Analysis

Participant demographics were summarized using descriptive statistics. Median assessment scores were compared pre‐ and post‐program using the Wilcoxon signed‐rank test. Statistical significance was defined as *p* < 0.05.

## Results

### Demographics

Of the 42 pwPD who were consented for participation, 25 (59.5%) completed all research obligations. The 17 participants who did not complete the study withdrew due to time constraints (*n* = 10), health deterioration or hospitalization (*n* = 2), family circumstances (*n* = 2), or were lost to follow‐up (*n* = 3). The median age was 70 years (IQR 67–73), 14 (56%) were female, and 24 (96%) were White. In addition, 14 (56%) were currently married or living with someone, while 11 (44%) lived alone. Median disease duration was 10 years (IQR 5–15) (Table 1).

**TABLE 1:** Demographics, persons with Parkinson's disease and students

	pwPD	Students
	(*n* = 25)	(*n* = 25)
Age, years; median (IQR)	70 (67–73)	20 (20–21)
Sex, *n* (%)		
Male	11 (44.0%)	10 (40.0%)
Female	14 (56.0%)	15 (60.0%)
Race, *n* (%)		
Asian	0 (0.0%)	14 (56.0%)
Black	1 (4.0%)	3 (12.0%)
Mixed	0 (0.0%)	2 (8.0%)
White	24 (96.0%)	6 (24.0%)
Marital status, *n* (%)		
Currently married, or living with someone in a marital‐like relationship	14 (56.0%)	0 (0.0%)
Never married & never lived with someone in a marital‐like relationship	3 (12.0%)	25 (100%)
Divorced or formerly lived with someone in a marital‐like relationship	4 (16.0%)	0 (0.0%)
Widowed	4 (16.0%)	0 (0.0%)
Disease duration, years; median (IQR)	10 (5–15)	‐‐
College year, *n* (%)		
Freshman	‐‐	1 (4.0%)
Sophomore	‐‐	9 (36.0%)
Junior	‐‐	8 (32.0%)
Senior	‐‐	7 (28.0%)
Field of study, *n* (%)		
STEM	‐‐	24 (96.0%)
Non‐STEM	‐‐	1 (4.0%)
Pre‐professional track, *n* (%)		
Pre‐medical	‐‐	20 (80.0%)
Non‐health profession	‐‐	5 (20.0%)
Family history of PD, *n* (%)		
Yes	‐‐	4 (16.0%)
No	‐‐	21 (84.0%)

Abbreviations: IQR, interquartile range; pwPD, persons with Parkinson's disease; STEM, Science, Technology, Engineering, or Mathematics.

The 25 students paired with participating pwPD also completed all obligations. The median age was 20 years (IQR 20–21), 15 (60%) were female, and 14 (56%) were Asian. Most students were upperclassmen (15, 60%) and pre‐medical (20, 80%). Only 4 (16%) reported a family history of PD (Table 1).

### Psychosocial Assessments

**Figure 1:**
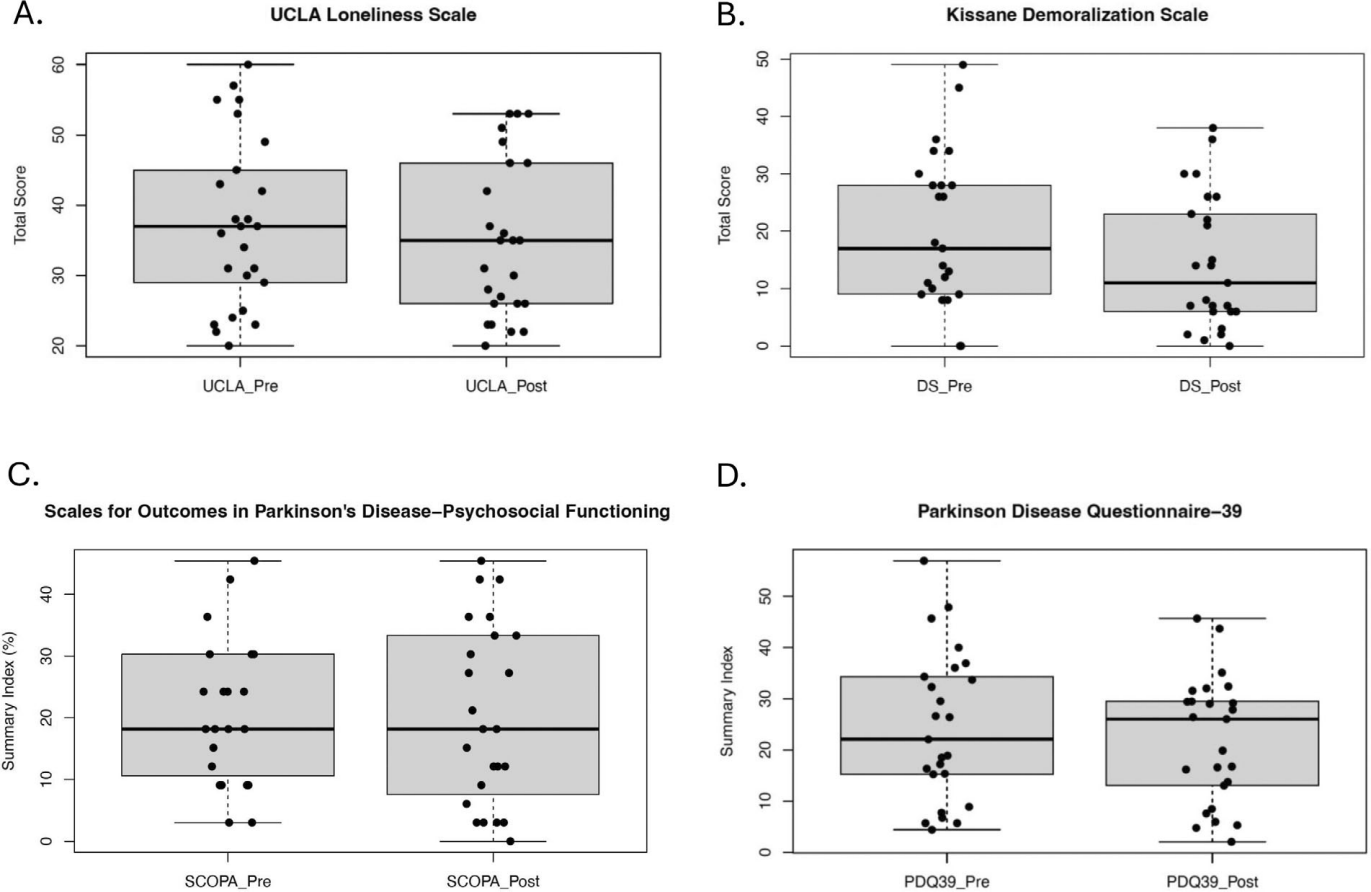
Total median pre‐program and post‐program scores. (A) Median score on the UCLA Loneliness Scale decreased from 37 pre‐program (IQR 29–45) to 35 (IQR 26–46) post‐program (*p* = 0.031), indicating less loneliness. (B) Median score on the Kissane Demoralization Scale (DS) decreased from 17 (IQR 9–28) pre‐program to 11 (IQR 6–23) post‐program (*p* < 0.001), indicating less demoralization. (C) Median summary index on the Scales for Outcomes in Parkinson's Disease‐Psychosocial Functioning (SCOPA‐PS) was 18.18% (IQR 9.1–30.3%) pre‐program and 18.18% (IQR 7.6–33.3%) post‐program (*p* = 0.445). (D) Median summary index on the Parkinson Disease Questionnaire‐39 (PDQ‐39) increased from 22.1 (IQR 15.3–34.3) pre‐program to 26.0 (IQR 13.1–29.5) post‐program (*p* = 0.141). Participants completed post‐program assessments approximately 1 week after program completion.

Median total UCLA‐LS score improved from 37 (IQR 29–45) pre‐program to 35 (IQR 26–46) post‐program (*p* = 0.031) (Fig. 1, Table S1). The percentage of pwPD experiencing moderate levels of loneliness decreased from 48% to 36%, while the percentage of pwPD with no/low levels of loneliness increased from 24% to 36%. High levels of loneliness remained stable at 25%.

Median total DS score improved from 17 (IQR 9–28) pre‐program to 11 (IQR 6–23) post‐program (*p* < 0.001) (Fig. 1, Table S2). The percentage of pwPD meeting the clinical threshold for demoralization (≥30) also decreased from 24% to 16%. Five specific domains showed statistically significant improvement: (1) “I am in good spirits” (*p* = 0.002), where the percentage reporting “always” increased from 16% to 40%; (2) “I feel irritable” (*p* = 0.011), where the percentage reporting “often” dropped from 40% to 16%; (3) “I cope fairly well with life” (*p* = 0.020), where the percentage reporting “always” increased from 12% to 40%; (4) “I am a worthwhile person” (*p* = 0.039), where the percentage reporting “always” increased from 44% to 64%; (5) “I feel trapped by what is happening to me” (*p* = 0.018), where the percentage reporting “never” increased from 52% to 60%.

Median summary index on the SCOPA‐PS was unchanged (difference = 0; *p* = 0.445), while median summary index on the PDQ‐39 increased (difference = +3.96; *p* = 0.141) but did not meet the MCID threshold (Fig. 1, Table S3).

### Student Knowledge and Post‐Program Feedback

When asked about their familiarity with PD, the percentage of students reporting “strongly agree” increased from 14.8% to 48.2% (*p* < 0.001) (Table S4). Both pwPD and students reported high levels of program satisfaction. When asked if they enjoyed the program, 84.0% of pwPD and 76.0% of students strongly agreed. When asked if virtual platforms were easy to navigate, 88% of pwPD and 92% of students agreed or strongly agreed. Three‐quarters of pwPD and students strongly recommended the program to others (Table S5).

## Discussion

In this unblinded feasibility study, participation in PD Pals was feasible and showed preliminary efficacy in reducing feelings of loneliness and demoralization among pwPD. In addition, students reported greater familiarity with PD. To our knowledge, this is the first study to examine the impact of a virtual intergenerational program on psychosocial symptoms in PD.

Loneliness and demoralization are well‐recognized in pwPD; however, few interventions target psychosocial symptoms. While some pwPD participate in peer support groups, these meetings may reinforce illness identity,^23^, ^24^ making PD Pals a unique way to form new relationships. In our study, observed improvements on the UCLA‐LS and DS were likely multifactorial. Regular contact with young adults provided pwPD with consistent social engagement to help counter the isolation experienced in later disease stages.^25^, ^26^ Unlike interactions with family members or healthcare providers, these relationships were voluntary and reciprocal, removing the burden of obligation or medical necessity.^14^ The intergenerational nature of these relationships also enhanced self‐worth, as pwPD assumed the role of a mentor and teacher by sharing life experiences and wisdom with student learners.^27^, ^28^ While we did not see improvement in SCOPA‐PS or PDQ‐39 scores, these non‐significant findings may be attributed to the small sample size or program duration. These metrics also included questions on mobility, cognition, and sexual function that would unlikely improve following PD Pals participation.

Program participation was also mutually beneficial for students. Direct interaction with pwPD provided experiential learning that classrooms cannot provide, giving students a firsthand understanding of disease heterogeneity. The longitudinal nature of the program also allowed students to observe and discuss PD symptoms and adaptive strategies over time. Based on positive program feedback, participation in PD Pals or similar intergenerational programs may inspire pre‐medical students to pursue careers in geriatric medicine or related fields.

PD Pals differs from prior intergenerational programs.^29^, ^30^, ^31^ Validated psychosocial assessments were used to determine the impact of program participation on pwPD. In addition, the virtual delivery model enabled broader geographic reach, eliminated transportation barriers, and allowed conversations to occur in comfortable home environments where participants had access to personal items and photographs that enhanced conversations. Pairing pwPD with undergraduate rather than medical students also expanded the participant pool and likely reduced the medical focus of shared conversations, allowing for more natural interactions.

These findings have important clinical implications. Psychosocial symptoms are common but rarely addressed in clinical settings until formal criteria for anxiety or depression are met, limiting opportunities for early intervention.^32^, ^33^, ^34^ Virtual intergenerational programs are low‐risk and cost‐effective, making them more suitable for broad implementation than traditional mental health services. PD Pals remains active at multiple universities in the United States, and we believe that further expansion is feasible, particularly if students partner with local PD centers.

There are several important limitations. First, the single‐center design, small sample size, and predominately White patient cohort limit generalizability, underscoring the need for targeted recruitment strategies and multi‐center validation.^35^ Next, lack of a designated control group prevents definitive causal attribution of observed findings, and a future randomized controlled trial is essential for determining program efficacy. Third, long‐term follow‐up data is needed to clarify whether observed benefits persist beyond the immediate post‐program period and whether additional improvements accrue with extended participation. Lastly, while no enrolled patients had dementia, we did not collect data on cognitive status, which may provide more insight into participant engagement and program benefit. Future studies should incorporate cognitive screening tools to better characterize the patient population and contextualize results.

## Conclusion

As evidenced by PD Pals, virtual intergenerational relationships may improve loneliness and demoralization in pwPD while also providing valuable learning experiences for undergraduate students. These findings warrant further investigation and highlight the potential for scalable, intergenerational programs to address unmet psychosocial needs in pwPD.

## Author Roles

(1) Research project: A. Conception, B. Organization, C. Execution; (2) Statistical Analysis: A. Design, B. Execution, C. Review and Critique; (3) Manuscript Preparation: A. Writing of the first draft, B. Review and Critique.

JG: 1A, 1B, 1C, 2C, 3A, 3B.

UG: 1A, 1B, 1C, 3B.

OG: 1A, 1B, 1C, 3A, 3B.

LE: 1C, 3B.

WA: 1A, 1B, 1C, 2A, 2B, 2C, 3A, 3B.

## Disclosure

**Ethical Compliance Statement:** This study was approved by the University of Pennsylvania Institutional Review Board (#854360). Written informed consent was obtained from all program participants. We confirm that we have read the Journal's position on issues involved in ethical publication and affirm that this work is consistent with those guidelines.

**Funding Sources and Conflicts of Interest:** This research was supported by the National Institutes of Health (1K23AG086669–01) and a College Alumni Society Research Grant from the University of Pennsylvania. Authors report no other disclosures or conflicts of interest relevant to this work.

**Financial Disclosures for the Previous 12 Months:** WWA received funding support from the National Institutes of Health (1K23AG086669‐01) and honoraria from Elsevier. JG, UG, and OHG received funding from the University of Pennsylvania College Alumni Society Research Grant, and LE reports no disclosures.

## Supporting information


**TABLE S1.** University of California Los Angeles (UCLA) Loneliness Scale Results. IQR; interquartile range. ^a^A 20‐item scale designed to measure one's subjective feelings of loneliness as well as feelings of social isolation. Participants rate each item on a scale from 1 (Never), 2 (Rarely), and 3 (Sometimes) to 4 (Often). Questions marked with an asterisk (*) are reverse scored. Total score for each participant is calculated by summing all responses for a score ranging from 20 to 80. ^b^Median score pre‐ and post‐intervention was compared using the Wilcoxon signed‐rank test. Statistically significant values are bolded.

**TABLE S2.** Kissane Demoralization Scale (DS) Results. IQR; interquartile range. ^a^ The DS, a 24‐item questionnaire, assesses the intensity and dimensions of demoralization. To score it, each item is rated on a 5‐point Likert scale (0–4), with higher scores indicating greater demoralization. Questions marked with an asterisk (*) are reverse scored. A total score is obtained by summing the individual item scores. ^b^Median score pre‐ and post‐intervention was compared using the Wilcoxon signed‐rank test. Statistically significant values are bolded.

**TABLE S3.** Scales for Outcomes in Parkinson's Disease‐Psychosocial Functioning (SCOPA‐PS) and Parkinson Disease Questionnaire‐39 (PDQ‐39) Results. IQR; interquartile range. ^a^Median score pre‐ and post‐intervention was compared using the Wilcoxon signed‐rank test. Statistically significant values are bolded. ^b^The SCOPA‐PS is a self‐administered, 11‐item questionnaire assessing psychosocial functioning during the preceding month on a scale ranging from 0 (not at all) to 3 (very much). A summary index is calculated by transforming the item sum score into a percentage of the maximum possible score (33 points). The higher the summary index, the worse the psychosocial functioning. ^c^The PDQ‐39 is scored by assigning a value from 0 to 4 for each of the 39 questions, with 0 representing “Never” and 4 representing “Always.” These scores are then used to calculate scores for eight different domains (mobility, daily activities, emotional well‐being, stigma, social support, cognition, communication, and bodily discomfort) and a summary index (sum of dimension total scores divided by 8). Higher scores indicate worse quality of life.

**TABLE S4.** Student Knowledge Areas. ^a^Responses were recorded on a 5‐point Likert scale, with 1 = strongly disagree, 2 = disagree, 3 = neutral, 4 = agree, and 5 = strongly agree. ^b^ Median Likert score was compared between groups using the Wilcoxon‐signed rank. Statistically significant values are highlighted in bold.

**TABLE S5.** Post‐Program Satisfaction Survey, Persons with Parkinson's Disease and Students. PwPD, persons with Parkinson's disease. ^a^Responses were recorded on a 5‐point Likert scale, with 1 = strongly disagree, 2 = disagree, 3 = neutral, 4 = agree, and 5 = strongly agree.

**Data S1.** Supplemental Methods: Supplement. A file of definitions explaining the assessments and measures used in the study along with references.

## Data Availability

The data that support the findings of this study are available from the corresponding author upon reasonable request.
