# Supplementary material for: The Impact of Participation in the Parkinson's Pals Program on Psychosocial Symptoms in Parkinson's Disease: An Unblinded Feasibility Study
Source: Mov Disord Clin Pract. 2026 Apr 1;13(9):2204–9. doi: 10.1002/mdc3.70589 (PMC13267603; doi:10.1002/mdc3.70589)
Supplement: Supplementary file 1 [file MDC3-13-2204-s001.docx]

**Supplemental Table 1: University of California Los Angeles (UCLA) Loneliness Scale Results**

| **UCLA Loneliness Scale Question^a^** | **Pre-Program**  **(Median, IQR)** | **Post-Program**  **(Median, IQR)** | **p-value^b^** |
| --- | --- | --- | --- |
| 1. How often do you feel that you are "in tune" with the people around you?* | 1 (1-2) | 1 (1-2) | 0.999 |
| 2. How often do you feel that you lack companionship? | 2 (1-3) | 2 (1-3) | 0.445 |
| 3. How often do you feel that there is no one you can turn to? | 1 (1-2) | 1 (1-2) | 0.511 |
| 4. How often do you feel alone? | 2 (1-3) | 2 (1-3) | 0.289 |
| 5. How often do you feel part of a group of friends?* | 2 (1-3) | 2 (1-2) | 0.121 |
| 6. How often do you feel that you have a lot in common with the people around you?* | 2 (1-2) | 2 (1-2) | 0.781 |
| 7. How often do you feel that you are no longer close to anyone? | 1 (1-2) | 1 (1-2) | 0.291 |
| 8. How often do you feel that your interests and ideas are not shared by those around you? | 2 (1-3) | 2 (2-3) | 0.999 |
| 9. How often do you feel outgoing and friendly?* | 2 (1-2) | 1 (1-2) | 0.577 |
| 10. How often do you feel close to people?* | 2 (1-2) | 2 (1-2) | 0.999 |
| 11. How often do you feel left out? | 2 (1-2) | 2 (1-3) | 0.973 |
| 12. How often do you feel that your relationships with others are not meaningful? | 2 (1-3) | 1 (1-2) | 0.188 |
| 13. How often do you feel that no one really knows you well? | 2 (1-3) | 2 (1-3) | 0.227 |
| 14. How often do you feel isolated from others? | 2 (1-3) | 2 (1-3) | 0.919 |
| 15. How often do you feel you can find companionship when you want it?* | 2 (1-2) | 1 (1-2) | 0.483 |
| 16. How often do you feel that there are people who really understand you?* | 2 (1-2) | 1 (1-2) | 0.125 |
| 17. How often do you feel shy? | 2 (2-3) | 2 (1-3) | 0.404 |
| 18. How often do you feel that people are around you but not with you? | 2 (2-3) | 2 (1-3) | 0.613 |
| 19. How often do you feel that there are people you can talk to?* | 1 (1-2) | 1 (1-2) | 0.344 |
| 20. How often do you feel that there are people you can turn to?* | 1 (1-2) | 1 (1-2) | 0.999 |
| Total Score | **37 (29-45)** | **35 (26-46)** | **0.031** |

Abbreviations: IQR; interquartile range

^a.^ A 20-item scale designed to measure one’s subjective feelings of loneliness as well as feelings of social isolation. Participants rate each item on a scale from 1 (Never), 2 (Rarely), and 3 (Sometimes) to 4 (Often). Questions marked with an asterisk (*) are reverse scored. Total score for each participant is calculated by summing all responses for a score ranging from 20 to 80.

^b.^ Median score pre- and post-intervention was compared using the Wilcoxon signed-rank test. Statistically significant values are bolded.
