# Supplementary material for: The Impact of Participation in the Parkinson's Pals Program on Psychosocial Symptoms in Parkinson's Disease: An Unblinded Feasibility Study
Source: Mov Disord Clin Pract. 2026 Apr 1;13(9):2204–9. doi: 10.1002/mdc3.70589 (PMC13267603; doi:10.1002/mdc3.70589)
Supplement: Supplementary file 5 [file MDC3-13-2204-s005.docx]

**Supplemental Table 5. Post-Program Satisfaction Survey, Persons with Parkinson’s Disease and Students**

| **Question** | **Response^a^** | **PwPD** | **Students** |
| --- | --- | --- | --- |
| I enjoyed the Parkinson’s Pals Program. | Strongly Agree  Agree  Neutral  Disagree  Strongly Disagree | 21 (84.0%)  4 (16.0%)  0 (0.0%)  0 (0.0%)  0 (0.0%) | 19 (76.0%)  6 (24.0%)  0 (0.0%)  0 (0.0%)  0 (0.0%) |
| I found it easy to navigate Zoom and meet with my Pal virtually. | Strongly Agree  Agree  Neutral  Disagree  Strongly Disagree | 15 (60.0%)  7 (28.0%)  1 (4.0%)  2 (8.0%)  0 (0.0%) | 12 (48.0%)  11 (44.0%)  2 (8.0%)  0 (0.0%)  0 (0.0%) |
| I would recommend the Parkinson’s Pals Program to others. | Strongly Agree  Agree  Neutral  Disagree  Strongly Disagree | 19 (76.0%)  6 (24.0%)  0 (0.0%)  0 (0.0%)  0 (0.0%) | 18 (72.0%)  7 (28.0%)  0 (0.0%)  0 (0.0%)  0 (0.0%) |

Abbreviations: PwPD, persons with Parkinson’s disease

^a^ Responses were recorded on a 5-point Likert scale, with 1=strongly disagree, 2=disagree, 3=neutral, 4=agree, and 5=strongly agree.
