# Supplementary material for: The Impact of Participation in the Parkinson's Pals Program on Psychosocial Symptoms in Parkinson's Disease: An Unblinded Feasibility Study
Source: Mov Disord Clin Pract. 2026 Apr 1:10.1002/mdc3.70589. Online ahead of print. doi: 10.1002/mdc3.70589 (PMC13267603; doi:10.1002/mdc3.70589)
Supplement: Supplementary file 2 — TABLE S2. Kissane Demoralization Scale (DS) Results. IQR; interquartile range. a The DS, a 24‐item questionnaire, assesses the intensity and dimensions of demoralization. To score it, each item is rated on a 5‐point Likert scale (0–4), with higher scores indicating greater demoralization. Questions marked with an asterisk (*) are reverse scored. A total score is obtained by summing the individual item scores. bMedian score pre‐ and post‐intervention was compared using the Wilcoxon signed‐rank test. Statistically significant values are bolded. [file MDC3-9999-0-s003.docx]

**Supplemental Table 2: Kissane Demoralization Scale (DS) Results**

| **DS Question^a^** | **Pre-Program**  **(Median, IQR)** | **Post-Program**  **(Median, IQR)** | **p-value^b^** |
| --- | --- | --- | --- |
| 1. There is a lot of value in what I can offer others.* | 1 (1-2) | 1 (0-2) | 0.380 |
| 2. My life seems to be pointless. | 0 (0-1) | 0 (0-1) | 0.727 |
| 3. There is no purpose to the activities in my life. | 0 (0-1) | 0 (0-1) | 0.273 |
| 4. My role in life has been lost. | 0 (0-1) | 0 (0-1) | 0.219 |
| 5. I no longer feel emotionally in control. | 1 (0-1) | 0 (0-1) | 0.231 |
| 6. I am in good spirits.* | 1 (1-1) | 1 (0-1) | **0.002** |
| 7. No one can help me. | 0 (0-1) | 0 (0-1) | 0.148 |
| 8. I feel that I cannot help myself. | 1 (0-1) | 0 (0-1) | 0.508 |
| 9. I feel hopeless. | 0 (0-1) | 0 (0-1) | 0.688 |
| 10. I feel guilty. | 0 (0-1) | 0 (0-1) | 0.366 |
| 11. I feel irritable. | 1 (1-2) | 1 (0-1) | **0.011** |
| 12. I cope fairly well with life.* | 1 (1-1) | 1 (0-1) | **0.020** |
| 13. I have a lot of regret about my life. | 0 (0-1) | 0 (0-1) | 0.375 |
| 14. Life is no longer worth living. | 0 (0-0) | 0 (0-0) | 0.125 |
| 15. I tend to feel hurt easily. | 1 (0-2) | 0 (0-1) | 0.148 |
| 16. I am angry about a lot of things. | 1 (0-2) | 0 (0-1) | 0.079 |
| 17. I am proud of my accomplishments.* | 1 (0-2) | 1 (0-2) | 0.999 |
| 18. I feel distressed about what is happening to me. | 2 (1-2) | 1 (0-2) | 0.093 |
| 19. I am a worthwhile person.* | 1 (0-2) | 0 (0-1) | **0.039** |
| 20. I would rather not be alive. | 0 (0-1) | 0 (0-0) | 0.313 |
| 21. I feel sad and miserable. | 1 (0-1) | 1 (0-1) | 0.999 |
| 22. I feel discouraged about life. | 1 (0-1) | 0 (0-1) | 0.289 |
| 23. I feel quite isolated or alone. | 1 (0-2) | 0 (0-1) | 0.188 |
| 24. I feel trapped by what is happening to me. | 0 (0-2) | 0 (0-1) | **0.018** |
| Total Score | **17 (IQR 9-28)** | **11 (IQR 6-23)** | **<0.001** |

Abbreviations: IQR; interquartile range

^a.^ The DS, a 24-item questionnaire, assesses the intensity and dimensions of demoralization. To score it, each item is rated on a 5-point Likert scale (0-4), with higher scores indicating greater demoralization. Questions marked with an asterisk (*) are reverse scored. A total score is obtained by summing the individual item scores.

^b.^ Median score pre- and post-intervention was compared using the Wilcoxon signed-rank test. Statistically significant values are bolded.
