# Supplementary material for: The Impact of Participation in the Parkinson's Pals Program on Psychosocial Symptoms in Parkinson's Disease: An Unblinded Feasibility Study
Source: Mov Disord Clin Pract. 2026 Apr 1:10.1002/mdc3.70589. Online ahead of print. doi: 10.1002/mdc3.70589 (PMC13267603; doi:10.1002/mdc3.70589)
Supplement: Supplementary file 3 — TABLE S3. Scales for Outcomes in Parkinson's Disease‐Psychosocial Functioning (SCOPA‐PS) and Parkinson Disease Questionnaire‐39 (PDQ‐39) Results. IQR; interquartile range. aMedian score pre‐ and post‐intervention was compared using the Wilcoxon signed‐rank test. Statistically significant values are bolded. bThe SCOPA‐PS is a self‐administered, 11‐item questionnaire assessing psychosocial functioning during the preceding month on a scale ranging from 0 (not at all) to 3 (very much). A summary index is calculated by transforming the item sum score into a percentage of the maximum possible score (33 points). The higher the summary index, the worse the psychosocial functioning. cThe PDQ‐39 is scored by assigning a value from 0 to 4 for each of the 39 questions, with 0 representing “Never” and 4 representing “Always.” These scores are then used to calculate scores for eight different domains (mobility, daily activities, emotional well‐being, stigma, social support, cognition, communication, and bodily discomfort) and a summary index (sum of dimension total scores divided by 8). Higher scores indicate worse quality of life. [file MDC3-9999-0-s005.docx]

**Supplemental Table 3: Scales for Outcomes in Parkinson’s Disease-Psychosocial Functioning (SCOPA-PS) and Parkinson Disease Questionnaire-39 (PDQ-39) Results**

| **Survey** | **Pre-Program Score**  **(Median, IQR)** | **Post-Program Score**  **(Median, IQR)** | **p-value^a^** |
| --- | --- | --- | --- |
| **Scales for Outcomes in Parkinson's Disease- Psychosocial Functioning (SCOPA-PS)^b^** |  |  |  |
| 1. During the past month, have you had difficulty with work, household or other chores? | 1 (0-2) | 1 (0-1) | 0.507 |
| 2. During the past month, have you had difficulty with hobbies, sport or leisure activities? | 1 (0-1) | 1 (0-1) | 0.999 |
| 3. During the past month, have you felt uncertain in your contact with others? | 0 (0-1) | 0 (0-1) | 0.766 |
| 4. During the past month, have you had problems getting along with your partner, family or good friends? | 0 (0-0) | 0 (0-1) | 0.727 |
| 5. During the past month, have you had problems in the area of sexuality? | 0 (0-0) | 0 (0-0) | 0.999 |
| 6. During the past month, have you felt more house-bound than you would wish to be? | 1 (0-1) | 1 (0-2) | 0.754 |
| 7. To what extent have you had the feeling that you have had to ask others for help *too* often during the past month? | 1 (0-1) | 0 (0-1) | 0.922 |
| 8. To what extent have you felt isolated and lonely during the past month? | 1 (0-1) | 0 (0-1) | 0.125 |
| 9. During the past month, have you had difficulty when having a conversation? | 1 (0-1) | 1 (0-1) | 0.999 |
| 10. To what extent have you felt ashamed of your disease during the past month? | 0 (0-1) | 0 (0-1) | 0.999 |
| 11. During the past month, have you been concerned about the future? | 1 (1-2) | 1 (0-2) | 0.563 |
| Summary Index | 18.2%  (IQR 9.1-30.3%) | 18.2%  (IQR 7.6-33.3%) | 0.445 |
| **Parkinson’s Disease Questionnaire-39 (PDQ-39)^c^** |  |  |  |
| Domain 1: Mobility (questions 1-10) | 25.0  (10.0-62.5) | 25.0  (5.0-42.5) | 0.320 |
| Domain 2: Activities of Daily Living (questions 11-16) | 20.8  (4.2-29.2) | 16.7  (4.2-25.0) | 0.132 |
| Domain 3: Emotional Well-being | 16.7  (12.5-37.5) | 20.8  (8.3-29.2) | 0.460 |
| Domain 4: Stigma | 6.3  (0-18.8) | 6.3  (0-12.5) | 0.086 |
| Domain 5: Social Support | 8.3  (0-16.7) | 8.3  (0-25.0) | 0.939 |
| Domain 6: Cognition | 25.0  (18.8-37.5) | 25.0  (12.5-31.3) | 0.117 |
| Domain 7: Communication | 25  (0.0-33.3) | 16.7  (8.3-33.3) | 0.942 |
| Domain 8: Bodily Discomfort | 41.7  (16.7-58.3) | 33.3  (16.7-58.3) | 0.652 |
| Summary Index | 22.1  (IQR 15.3-34.3) | 26.0  (IQR 13.1-29.5) | 0.141 |

Abbreviations: IQR; interquartile range

^a.^ Median score pre- and post-intervention was compared using the Wilcoxon signed-rank test. Statistically significant values are bolded.

^b.^ The SCOPA-PS is a self-administered, 11-item questionnaire assessing psychosocial functioning during the preceding month on a scale ranging from 0 (not at all) to 3 (very much). A summary index is calculated by transforming the item sum score into a percentage of the maximum possible score (33 points). The higher the summary index, the worse the psychosocial functioning.

^c.^ The PDQ-39 is scored by assigning a value from 0 to 4 for each of the 39 questions, with 0 representing "Never" and 4 representing "Always". These scores are then used to calculate scores for eight different domains (mobility, daily activities, emotional well-being, stigma, social support, cognition, communication, and bodily discomfort) and a summary index (sum of dimension total scores divided by 8). Higher scores indicate worse quality of life.
