# Supplementary material for: The Impact of Participation in the Parkinson's Pals Program on Psychosocial Symptoms in Parkinson's Disease: An Unblinded Feasibility Study
Source: Mov Disord Clin Pract. 2026 Apr 1:10.1002/mdc3.70589. Online ahead of print. doi: 10.1002/mdc3.70589 (PMC13267603; doi:10.1002/mdc3.70589)
Supplement: Supplementary file 4 — TABLE S4. Student Knowledge Areas. aResponses were recorded on a 5‐point Likert scale, with 1 = strongly disagree, 2 = disagree, 3 = neutral, 4 = agree, and 5 = strongly agree. b Median Likert score was compared between groups using the Wilcoxon‐signed rank. Statistically significant values are highlighted in bold. [file MDC3-9999-0-s001.docx]

**Supplemental Table 4. Student Knowledge Areas**

| **Question** | **Response^a^** | **Pre-Program** | **Post-Program** | **p-value^b^** |
| --- | --- | --- | --- | --- |
| I have good oral communication skills. | Strongly Agree  Agree  Neutral  Disagree  Strongly Disagree | 15 (60.0%)  9 (36.0%)  1 (4.0%)  0 (0.0%)  0 (0.0%) | 14 (56.0%)  9 (36.0%)  2 (8.0%)  0 (0.0%)  0 (0.0%) | 0.625 |
| I feel comfortable interacting with older adults. | Strongly Agree  Agree  Neutral  Disagree  Strongly Disagree | 12 (48.0%)  12 (48.0%)  1 (4.0%)  0 (0.0%)  0 (0.0%) | 15 (60.0%)  10 (40.0%)  0 (0.0%)  0 (0.0%)  0 (0.0%) | 0.250 |
| I can learn from older adults. | Strongly Agree  Agree  Neutral  Disagree  Strongly Disagree | 21 (84.0%)  3 (12.0%)  1 (4.0%)  0 (0.0%)  0 (0.0%) | 22 (88.0%)  3 (12.0%)  0 (0.0%)  0 (0.0%)  0 (0.0%) | 0.625 |
| I feel comfortable interacting with older adults with chronic diseases. | Strongly Agree  Agree  Neutral  Disagree  Strongly Disagree | 9 (36.0%)  10 (40.0%)  6 (24.0%)  0 (0.0%)  0 (0.0%) | 12 (48.0%)  12 (48.0%)  1 (4.0%)  0 (0.0%)  0 (0.0%) | 0.056 |
| I am familiar with Parkinson’s disease and related disorders. | Strongly Agree  Agree  Neutral  Disagree  Strongly Disagree | 4 (16.0%)  14 (56.0%)  3 (12.0%)  4 (16.0%)  0 (0.0%) | 11 (44.0%)  13 (52.0%)  1 (4.0%)  0 (0.0%)  0 (0.0%) | **<0.001** |
| Parkinson’s disease can impact a patient’s social relationships and functioning. | Strongly Agree  Agree  Neutral  Disagree  Strongly Disagree | 17 (68.8%)  7 (28.0%)  1 (4.0%)  0 (0.0%)  0 (0.0%) | 17 (68.0%)  5 (20.0%)  2 (8.0%)  1 (4.0%)  0 (0.0%) | 0.766 |
| True or False. Parkinson’s disease has little variability and most patients look identical. | True  False | 0 (0%)  25 (100%) | 0 (0%)  25 (100%) | 1.000 |
| True or False. Parkinson’s medication can slow disease progression. | True  False | 11 (44.0%)  14 (56.0%) | 13 (52.0%)  12 (48.0%) | 0.727 |

^a^ Responses were recorded on a 5-point Likert scale, with 1=strongly disagree, 2=disagree, 3=neutral, 4=agree, and 5=strongly agree.

^b^ Median Likert score was compared between groups using the Wilcoxon signed rank. Statistically significant values are highlighted in **bold.**
