# Supplementary material for: The Impact of Participation in the Parkinson's Pals Program on Psychosocial Symptoms in Parkinson's Disease: An Unblinded Feasibility Study
Source: Mov Disord Clin Pract. 2026 Apr 1:10.1002/mdc3.70589. Online ahead of print. doi: 10.1002/mdc3.70589 (PMC13267603; doi:10.1002/mdc3.70589)
Supplement: Supplementary file 6 — Data S1. Supplemental Methods: Supplement. A file of definitions explaining the assessments and measures used in the study along with references. [file MDC3-9999-0-s002.docx]

**Supplement**

The UCLA Loneliness Scale Version 3 is a 20-item scale designed to measure subjective feelings of loneliness and social isolation.^1^ Participants rate each question item on a scale from 1 (never) to 4 (often). The total score is calculated by summing all responses for each participant, with a final score ranging from 20 to 80. Higher scores indicate greater loneliness. The loneliness categories are defined as follows: total score <28 = no/low loneliness, total score 28-43 = moderate loneliness, total score >43 = high degree of loneliness. A minimally clinically important difference (MCID) has not been established.

The Kissane Demoralization Scale (DS) is a 24-item questionnaire that assesses the intensity and dimensions of demoralization.^2^ Each item is rated on a 5-point Likert scale (0-4), with higher scores indicating greater demoralization. A total score is obtained by summing the individual item scores. Scores are often interpreted in the context of clinical significance. For example, in the original validation study with cancer patients, scores of 30 or higher were considered to indicate clinically significant demoralization. While a MCID has not been established for the DS, a difference of 2 points between groups on the refined Demoralization Scale-II (DS-II), a 16-item, self-reported measure of demoralization, is considered clinically meaningful.^3^

The SCales for Outcomes in PArkinson's disease- Psychosocial Functioning (SCOPA-PS) is a self-administered, 11-item questionnaire assessing psychosocial functioning during the preceding month, on a scale ranging from 0 (not at all) to 3 (very much).^4^ A summary index is calculated by transformation of the item sum score into a percentage of the maximum possible score (33 points). The higher the summary index, the worse the psychosocial functioning. In one study, the minimally important change (MIC) value for patients who experienced a real worsening in their health status was 8.30-9.10, though the PDQ-39 was used as the reference and other anchors may produce different responsiveness and MIC values.^5^

The Parkinson’s Disease Questionnaire-39 (PDQ-39) is scored by assigning a value from 0 to 4 for each of the 39 questions, with 0 representing “never” and 4 representing “always”.^6^ These scores are then used to calculate scores for eight different domains (mobility, daily activities, emotional well-being, stigma, social support, cognition, communication, and bodily discomfort) and a summary index. Higher scores indicate worse quality of life. The domain score is determined by the sum of scores of each item in the dimension divided by the maximum possible score of all the items in the dimension, multiplied by 100. The overall score can be summarized in the Parkinson’s Disease Summary Index (PDSI) or PDQ-39 Summary Index (PDQ-39 SI). PDSI or PDQ-39 SI = sum of dimension total scores divided by 8. The most optimal estimates for MCID thresholds on the PDQ-39 SI are -4.72 and +4.22 for detecting minimal clinically important improvement and worsening.^7^

**References:**

1. Russell DW. UCLA Loneliness Scale (Version 3): reliability, validity, and factor structure. *J Pers Assess*. 1996;66(1):20-40. doi:10.1207/s15327752jpa6601_2

2. Kissane DW, Wein S, Love A, Lee XQ, Kee PL, Clarke DM. The Demoralization Scale: a report of its development and preliminary validation. *J Palliat Care*. 2004;20(4):269-276.

3. Robinson S, Kissane DW, Brooker J, et al. Refinement and revalidation of the demoralization scale: The DS-II-external validity. *Cancer*. 2016;122(14):2260-2267. doi:10.1002/cncr.30012

4. Marinus J, Visser M, Martínez-Martín P, van Hilten JJ, Stiggelbout AM. A short psychosocial questionnaire for patients with Parkinson’s disease: the SCOPA-PS. *J Clin Epidemiol*. 2003;56(1):61-67. doi:10.1016/s0895-4356(02)00569-3

5. Martínez-Martin P, Carod-Artal FJ, da Silveira Ribeiro L, et al. Longitudinal psychometric attributes, responsiveness, and importance of change: An approach using the SCOPA-Psychosocial questionnaire. *Mov Disord*. 2008;23(11):1516-1523. doi:10.1002/mds.22202

6. Jenkinson C, Fitzpatrick R, Peto V, Greenhall R, Hyman N. The Parkinson’s Disease Questionnaire (PDQ-39): development and validation of a Parkinson’s disease summary index score. *Age Ageing*. 1997;26(5):353-357. doi:10.1093/ageing/26.5.353

7. Horváth K, Aschermann Z, Kovács M, et al. Changes in Quality of Life in Parkinson’s Disease: How Large Must They Be to Be Relevant? *Neuroepidemiology*. 2017;48(1-2):1-8. doi:10.1159/000455863
